# Incidence and relative risk of cutaneous squamous cell carcinoma with single-agent BRAF inhibitor and dual BRAF/MEK inhibitors in cancer patients: a meta-analysis

**DOI:** 10.18632/oncotarget.21059

**Published:** 2017-09-19

**Authors:** Ling Peng, Yina Wang, Yun Hong, Xianghua Ye, Peng Shi, Junyan Zhang, Qiong Zhao

**Affiliations:** ^1^ Department of Thoracic Oncology, The First Affiliated Hospital, School of Medicine, Zhejiang University, Hangzhou, Zhejiang Province, China; ^2^ Department of Pharmacy, The First Affiliated Hospital, School of Medicine, Zhejiang University, Hangzhou, Zhejiang Province, China; ^3^ Department of Radiotherapy, The First Affiliated Hospital, School of Medicine, Zhejiang University, Hangzhou, Zhejiang Province, China; ^4^ Department of Medical Statistics, Children’s Hospital of Fudan University, Shanghai, China; ^5^ Center for Evidence-based Medicine, Fudan University, Shanghai, China; ^6^ Bothwin Clinical Study Consultant, Bellevue, WA, USA

**Keywords:** BRAF inhibitor, MEK inhibitor, cuSCC, meta-analysis

## Abstract

**Background:**

BRAF inhibitor and dual BRAF/MEK inhibitors have been approved for the treatment of BRAF-mutated melanoma. Cutaneous squamous cell carcinoma (cuSCC) is an adverse event associated with these drugs. The contribution of BRAF inhibitor and dual BRAF/MEK inhibitors to cuSCC are still unknown. We performed this meta-analysis to determine the overall incidence and relative risk of cuSCC in cancer patients treated with these drugs.

**Results:**

A total of 7,442 patients from 24 primary studies were included. The incidences of all-grade and high-grade cuSCC in cancer patients treated with BRAF inhibitor were 12.5% (95% CI: 10.8–14.6%) and 11.6% (95% CI: 9.8–13.8%), and dual BRAF/MEK inhibitors were 3.0% (95% CI: 2.0–4.5%) and 2.8% (95% CI: 1.9–4.0%), respectively. On subgroup analysis and meta-regression, the incidence of cuSCC did not vary with tumor type, study design and specific drug used. The use of single agent BRAF inhibitor significantly increased the risk of developing cuSCC comparing with dual BRAF/MEK inhibitors for all-grade (RR 4.72, 95% CI: 2.42–9.20) and high-grade (RR 4.92, 95% CI: 2.64–9.16) in cancer patients.

**Materials and Methods:**

The databases of PubMed, Embase and abstracts published in ASCO proceedings were searched for relevant studies from January 2000 to June 2017. Summary incidences, relative risks (RRs) and 95% confidence intervals (CIs) were calculated by using either random effects or fixed effect models according to the heterogeneity of included studies.

**Conclusions:**

BRAF inhibitor significantly increases the risk of developing cuSCC compared with dual BRAF/MEK inhibitors in cancer patients. Clinicians should be aware of the risks of cuSCC with the administration of these drugs in cancer patients.

## INTRODUCTION

The MAPK signaling pathway regulates cell growth, proliferation, and differentiation [1]. MAPK kinase (MEK) activation occurs downstream of RAS and RAF signaling, activating MAPK. Previous study found that mutations in BRAF can be found in 8% of human cancers, including 59% of melanomas, 30–70% of thyroid cancers, 30% of cancers of the ovary of low grade and 10% of colorectal cancers [2]. In recent years, targeted drugs against BRAF have shown remarkable therapeutic success for a subset of patients with advanced malignancies including melanoma, non-small cell lung cancer (NSCLC) and other malignancies, leading to their FDA approval. At present, its utility is being explored for other cancers in over 200 clinical trials.

New primary cutaneous malignancies, including cutaneous squamous cell carcinoma (cuSCC), basal cell carcinoma, keratoacanthoma and melanoma have been reported in patients receiving BRAF inhibitor, most of which are cuSCC. The exact molecular mechanisms behind the increased incidence are poorly understood. However, it is hypothesized that this may be due to RAF inhibition of wild-type BRAF cells, together with oncogenic RAS mutations present in photodamaged skin. This toxicity is explained in part by “paradoxical ERK activation,” or the hyperactivation of ERK signaling by BRAF inhibitor in BRAF wild-type cells.

Although cuSCC is not life-threatening, it may affect BRAF inhibitor dosing, and impact patients’ physical, social, and psychological well-being. The overall incidence and risk of cuSCC caused by BRAF inhibitor is yet to be well defined, due to the limited number of studies available, the variation of the results reported in each of these trials, as well as the differences in study-settings and tumor-stream. Therefore, we conducted a systematic literature search and meta-analysis to investigate the overall incidence and relative risk of developing cuSCC in patients receiving BRAF inhibitor and dual BRAF/MEK inhibitors. We have also explored potential risk factors including specific drug, tumor type and study design. This information is critical in the determination of therapeutic regimens, and contributes to efforts in understanding the underlying mechanisms, risk factors and devising evidence-based treatment strategies for cuSCC.

## RESULTS

### Study selection and characteristics

**Figure 1:**
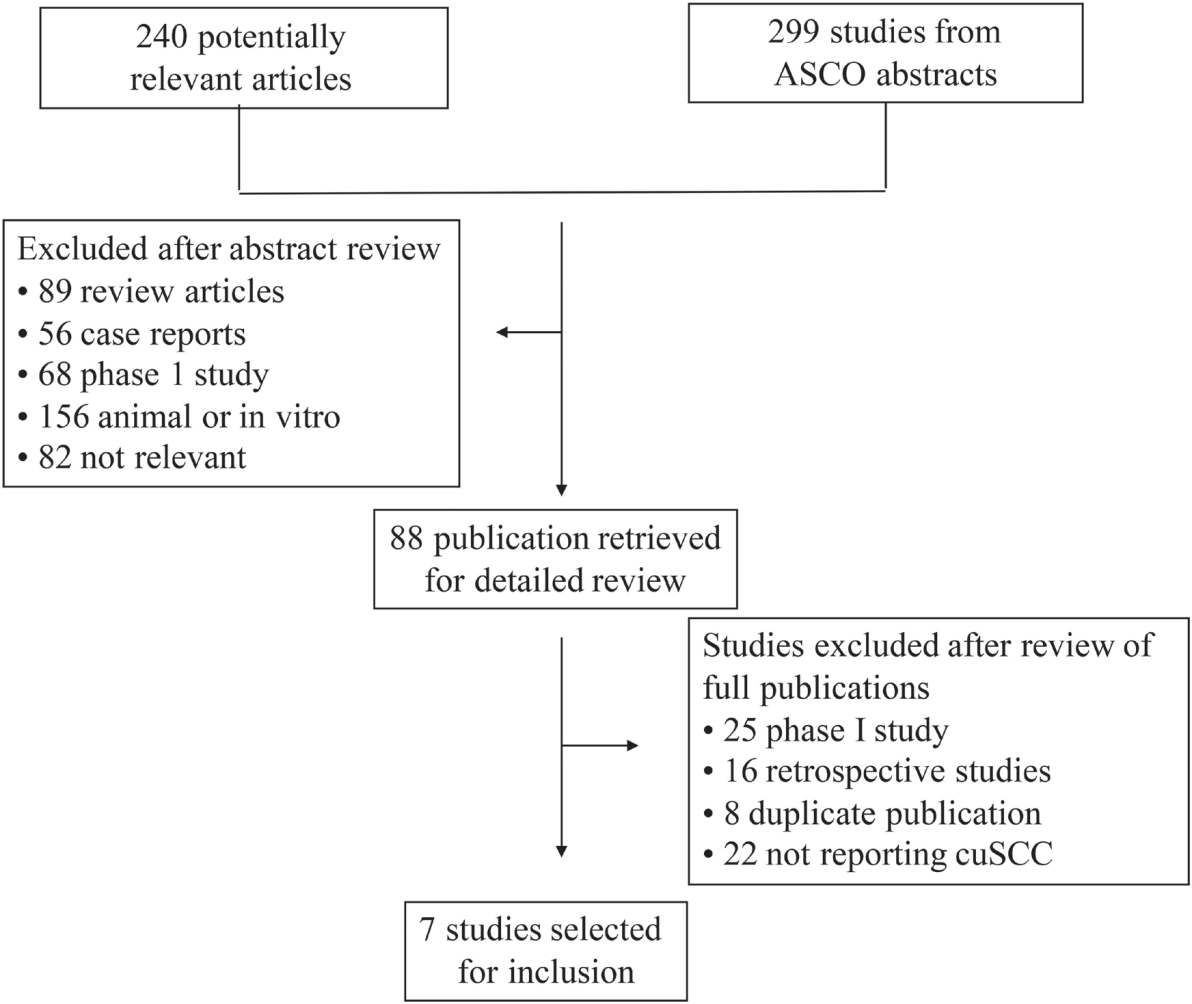
Flow diagram of selection process for the trials included in the meta-analysis

Our comprehensive search of the literature revealed 240 potentially relevant records (Figure 1). Among them, 218 were excluded during the selection process. Of the 22 studies included, 15 were non-randomized studies while the other 7 were randomized controlled trials. A total of 299 ASCO meeting abstracts were retrieved, however only 2 studies were found eligible. In all, 24 primary studies were shortlisted for statistical analysis (Table 1). The major baseline characteristics of the 24 eligible studies were reported in Table 1, encompassing 5 phase III randomized controlled trials, 17 phase II trials, and 2 phase IV trials. The sample size of the included studies ranged from 21 to 3,222 patients (median sample size, 132 patients). The studies were published between 2012 and 2017.

**Table 1:** Main characteristics and results of the eligible studies

Year	Study	Phase	Source	Disease	Trial	Calculation	Drug	All-grade	High-grade	Patients
2017	Subbiah [26]	2	ASCO	NSCLC	Single-arm	Incidence	Vemurafenib	NR	9	62
2017	McArthur [27]	2	Pubmed	Melanoma	Single-arm	Incidence	Vemurafenib	17	17	146
2016	Planchard 2 [28]	2	Pubmed	NSCLC	Single-arm	Incidence	Dabrafenib+Trametinib	2	2	59
2016	Planchard 1 [29]	2	Pubmed	NSCLC	Single-arm	Incidence	Dabrafenib	10	10	84
2016	Chen [30]	2	Pubmed	Melanoma	Single-arm	Incidence	Dabrafenib+Trametinib	0	0	23
2016	Brose [5]	2	Pubmed	Thyroid	Single-arm	Incidence	Vemurafenib	2	2	51
2016	Ascierto [11]	3	Pubmed	Melanoma	RCT	Incidence & RR	Vemurafenib+Cobimetinib	10	9	247
							Vemurafenib	31	31	246
2015	Tiacci [6]	2	Pubmed	Leukemia	Single-arm	Incidence	Vemurafenib	3	0	54
2015	Robert [13]	3	Pubmed	Melanoma	RCT	Incidence & RR	Dabrafenib+Trametinib	5	5	350
							Vemurafenib	63	60	349
2015	Puzanov [4]	2	Pubmed	Melanoma	Single-arm	Incidence	Vemurafenib	16	16	48
2015	Long [12]	3	Pubmed	Melanoma	RCT	Incidence & RR	Dabrafenib+Trametinib	6	6	209
							Dabrafenib	20	20	211
2015	Kopetz [31]	2	Pubmed	Colorectal	Single-arm	Incidence	Vemurafenib	5	5	21
2015	Hyman [32]	2	Pubmed	Nonmelanoma	Single-arm	Incidence	Vemurafenib	22	22	95
2014	McArthur [7]	3	Pubmed	Melanoma	RCT	Incidence	Vemurafenib	65	65	337
2014	Larkin [33]	4	Pubmed	Melanoma	Single-arm	Incidence	Vemurafenib	437	389	3222
2014	Flaherty [34]	4	Pubmed	Melanoma	Single-arm	Incidence	Vemurafenib	22	10	371
2014	Dummer [35]	2	Pubmed	Melanoma	Single-arm	Incidence	Vemurafenib	4	4	24
2013	Ascierto [36]	2	Pubmed	Melanoma	Single-arm	Incidence	Dabrafenib	9	7	92
2012	Sosman [37]	2	Pubmed	Melanoma	Single-arm	Incidence	Vemurafenib	34	34	132
2012	Long [38]	2	Pubmed	Melanoma	Single-arm	Incidence	Dabrafenib	11	11	172
2012	Lebbe [39]	2	ASCO	Melanoma	Single-arm	Incidence	Vemurafenib	20	20	507
2012	Hauschild [8]	3	Pubmed	Melanoma	RCT	Incidence	Dabrafenib	12	8	187
2012	Flaherty [40]	2	Pubmed	Melanoma	RCT	Incidence & RR	Dabrafenib+Trametinib	5	4	109
							Dabrafenib	10	9	53
2012	Anforth [24]	2	Pubmed	Melanoma	Single-arm	Incidence	Dabrafenib	8	NR	43

Summary table of studies included in the meta-analysis. Abbreviations: N, number of patients; CI, confidence interval; NR, not reported.

A total of 7,442 patients with underlying malignancy diagnoses of melanoma (17 trials), NSCLC (3 trials), colorectal (1 trial), thyroid (1 trial), leukemia (1 trial), and other non-melanoma malignancy (1 trial) were available for the meta-analysis. Of these, 6,445 patients received BRAF inhibitor as a single agent (vemurafenib 5,603; and dabrafenib 842). Only four trials were randomized controlled trials eligible for analysis of relative risk. None of the included studies had listed cuSCC as a pre-existing condition. We performed this meta-analysis in accordance with the guidelines of the Preferred Reporting Items for Systematic review and Meta-Analyses (PRISMA) statement [3].

### Incidence of cuSCC

**Figure 2:**
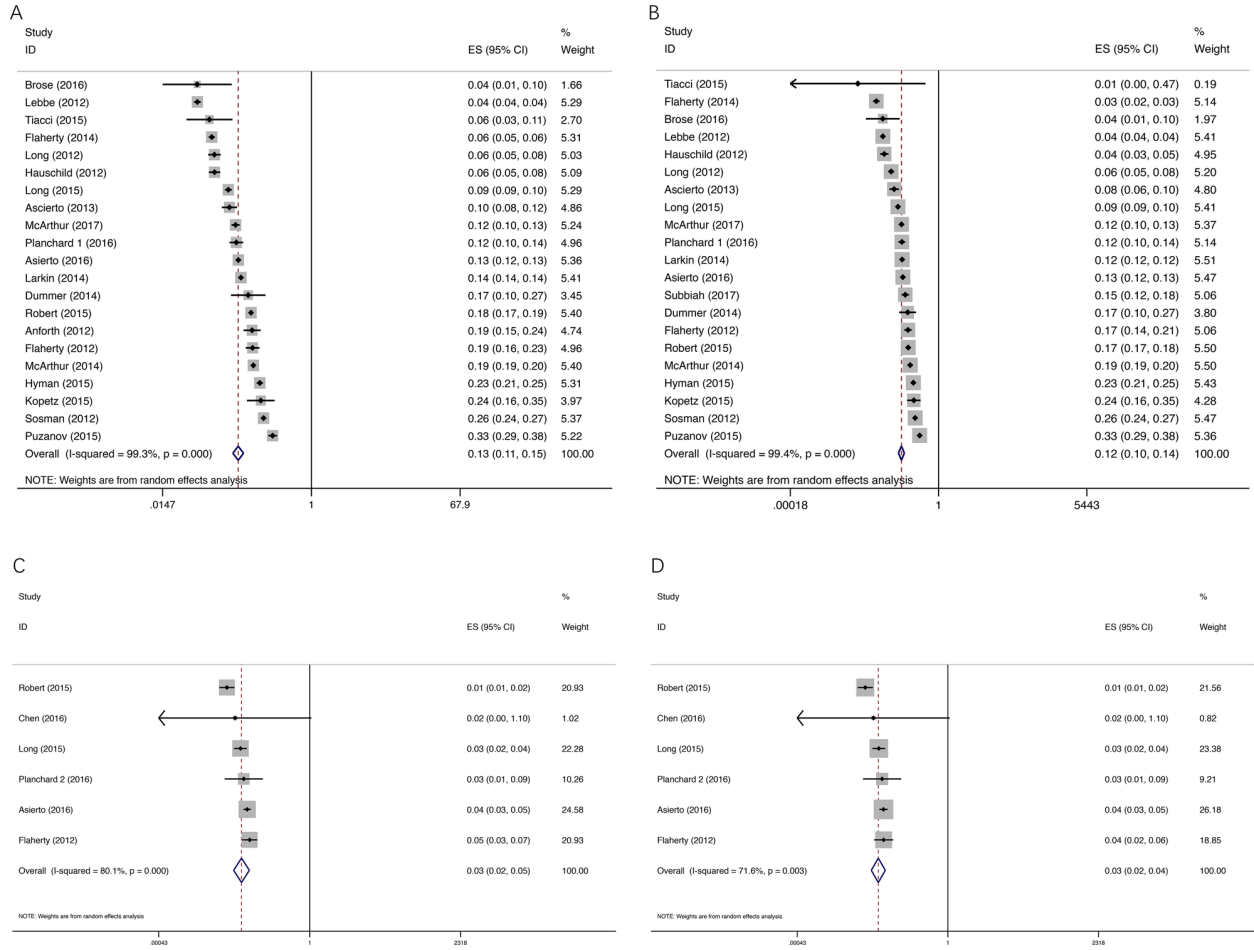
Forest plot for meta-analysis of incidence of all-grade and high-grade cuSCC Each study was shown by the name of the lead author and year of publication. The summary incidences were also shown in the figure. Plots are arranged as follows: (**A**) Incidence of all-grade cuSCC by BRAF inhibitor; (**B**) Incidence of high-grade cuSCC by BRAF inhibitor; (**C**) Incidence of all-grade cuSCC by dual BRAF/MEK inhibitors; (**D**) Incidence of high-grade cuSCC by dual BRAF/MEK inhibitors.

The results of the meta-analysis were shown in Figure 2. Overall, a total of 6,445 patients from 21 trials were included for analysis of all-grade and incidence of cuSCC. The incidence of all-grade cuSCC ranged from 3.92 to 33.33%; the lowest incidence was noted in a phase 2 trial by Puzanov *et al.* [4] in patients with melanoma, and the highest incidence was observed in patients with thyroid cancer [5]. Our meta-analysis revealed a significant heterogeneity among included studies (*I*^2^ = 99.33%, *P* < 0.0001), and the calculated summary incidence of all-grade cuSCC with BRAF inhibitor was 12.5% (95% CI: 10.8–14.6%) using a random effects model (Figure 2A). Twenty-one trials reported the incidence of high-grade cuSCC data ranging from 0 to 33.33%. The highest incidence was observed in a phase II trial conducted by Puzanov *et al.* [4], and the lowest incidence was observed in patients with leukemia [6]. The calculated summary incidence of high-grade cuSCC associated with BRAF inhibitor was 11.6% (95% CI: 9.8–13.8%), using a random effects model (Figure 2B).

As for dual BRAF/MEK inhibitors, the incidences of all-grade and high-grade cuSCC were lower than those of single agent BRAF inhibitor, with all-grade incidence of 3.0% (95% CI: 2.0–4.5%) and high-grade incidence of 2.8% (95% CI: 1.9–4.0%), respectively. Our meta-analysis revealed a significant heterogeneity among included studies (all grade, *I*^2^ = 80.1%, *P* < 0.0001; and high-grade, *I*^2^ = 71.6%, *P* = 0.003) (Figure 2).

**Figure 3:**
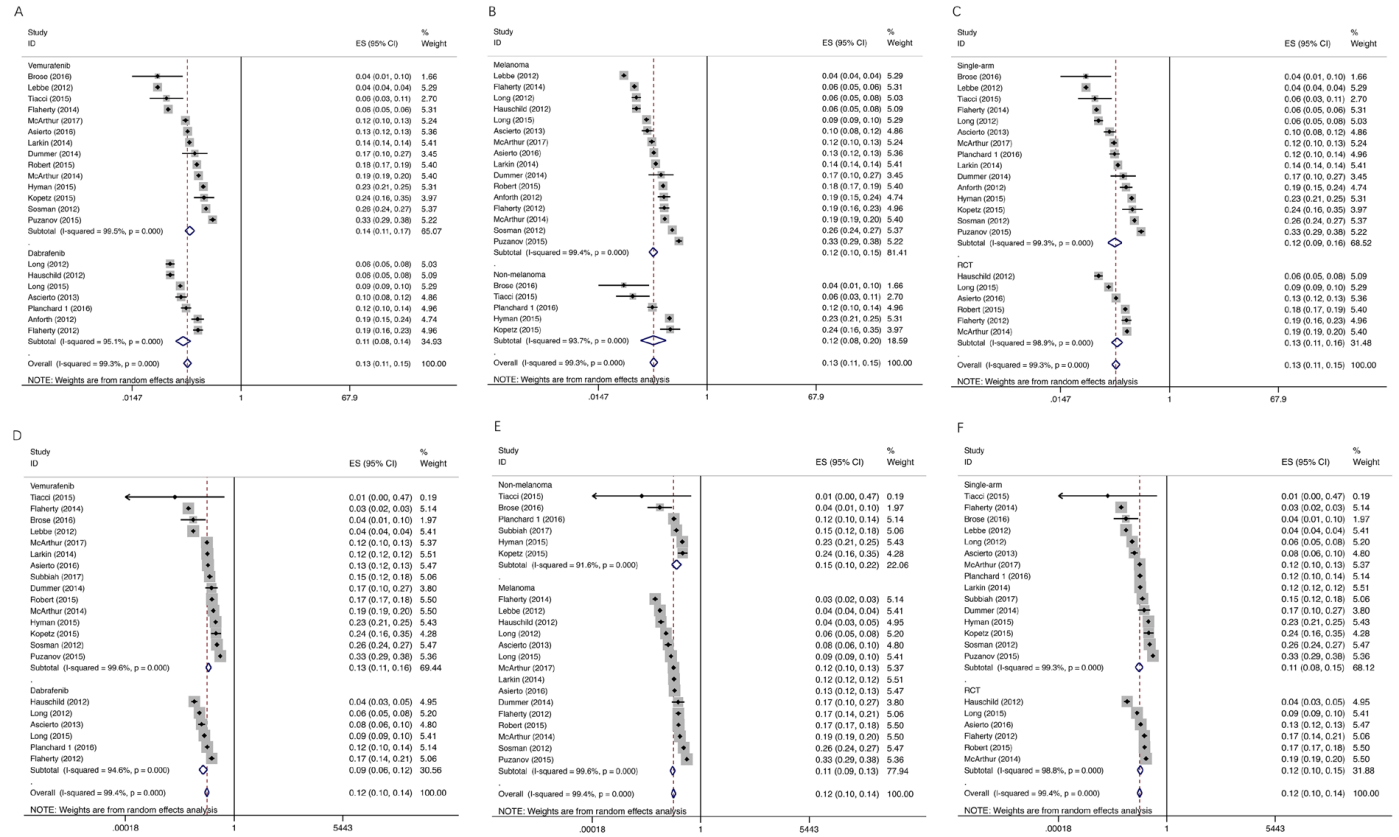
Subgroup analysis for incidence of all-grade and high-grade cuSCC Each study was shown by the name of the lead author and year of publication. The summary incidences were also shown in the figure. Plots are arranged as follows: (**A**) Incidence of all-grade cuSCC by vemurafenib vs dabrafenib subgroup; (**B**) Incidence of all-grade cuSCC in melanoma vs non-melanoma; (**C**) Incidence of all-grade cuSCC from single-arm study vs RCT; (**D**) Incidence of cuSCC by vemurafenib vs dabrafenib subgroup; (**E**) Incidence of high-grade cuSCC in melanoma vs non-melanoma; (**F**) Incidence of high-grade cuSCC from single-arm study vs RCT.

We conducted a meta regression analysis to examine whether incidence of cuSCC varied by specific BRAF inhibitor, melanoma versus non-melanoma, or study design. We found that there was no significant effect of these factors on the incidence for either all-grade or high-grade cuSCC (all *P* > 0.05). Additionally, there was no significant effect of these factors on the incidence of either all grade or high grade cuSCC by subgroup analysis (Figure 3).

### Relative risk of cuSCC

**Figure 4:**
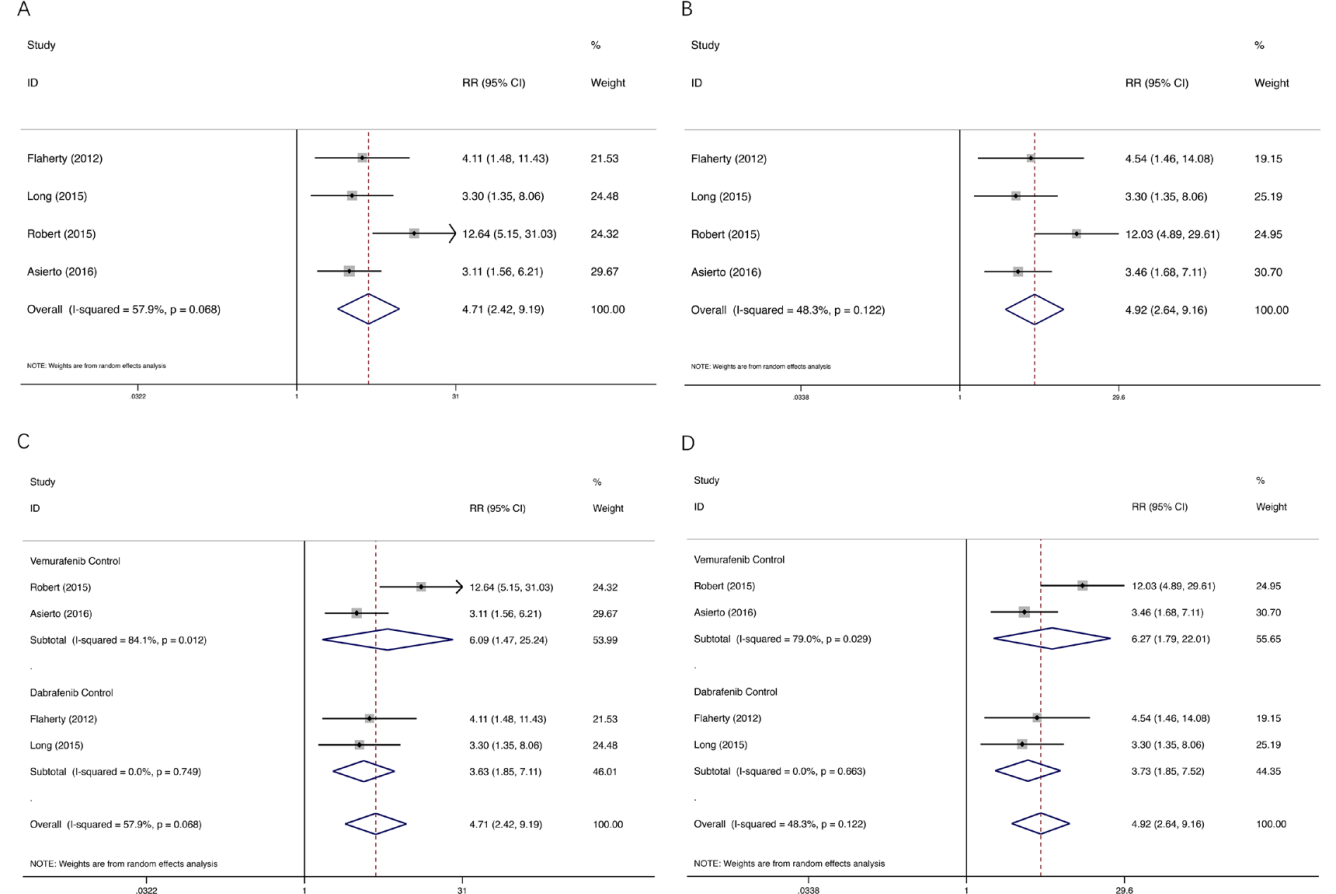
RR for all-grade and high-grade cuSCC Each study was shown by the name of the lead author and year of publication. The RRs were also shown in the figure. Plots are arranged as follows: (**A**) RR of all-grade cuSCC comparing BRAF inhibitor versus dual BRAF/MEK inhibitors; (**B**) RR of high-grade cuSCC comparing BRAF inhibitor versus dual BRAF/MEK inhibitors; (**C**) RR of all-grade cuSCC comparing BRAF inhibitor versus dual BRAF/MEK inhibitors by subgroup; (**D**) RR of high-grade cuSCC comparing BRAF inhibitor versus dual BRAF/MEK inhibitors by subgroup.

We determined the RR of BRAF inhibitor–induced cuSCC compared with dual BRAF/MEK inhibitors. Analysis of the 1,774 patients across 4 RCTs revealed that BRAF inhibitor increased the risk of developing all-grade and high-grade cuSCC in cancer patients with a RR of 4.72, 95% CI: 2.42–9.20, and RR of 4.92, 95% CI: 2.64–9.16, respectively (Figure 4), suggesting a nearly five-fold greater risk for developing cuSCC with single agent BRAF inhibitor versus dual BRAF/MEK inhibitors. Significant heterogeneity was found for all-grade (test for heterogeneity: *I*^2^ = 57.9%; *P* = 0.068), but not for high-grade (*I*^2^ = 48.3%; *P* = 0.122).

To evaluate the impact of the control regimen on the RR of cuSCC, studies have been classified into two subgroups; two studies with vemurafenib as monotherapy, and two studies with dabrafenib. There were no statistically significance between the subgroups (Figure 4).

### Publication bias

**Figure 5:**
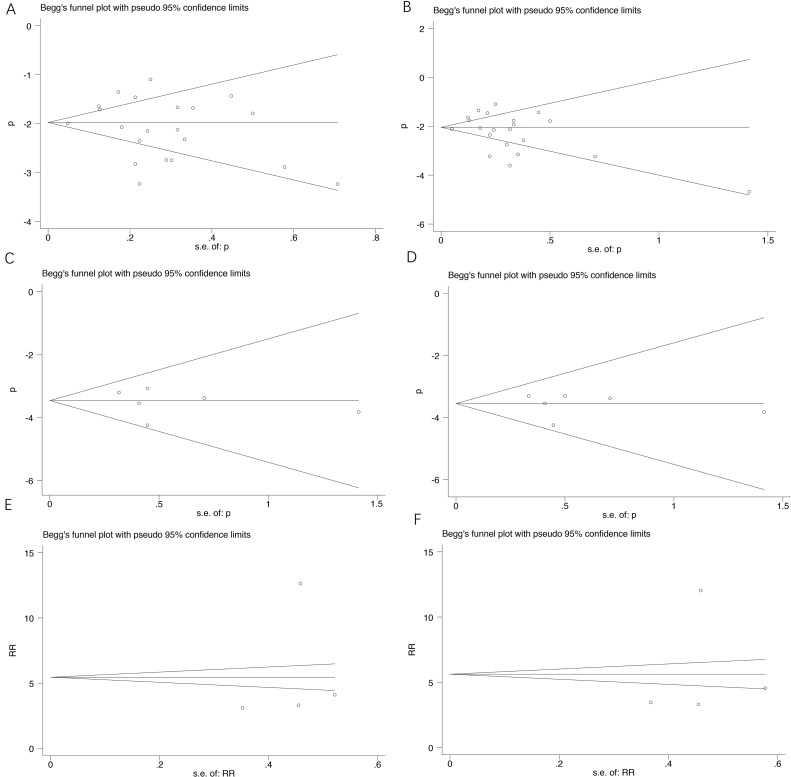
Funnel plot for studies included in the meta-analysis Each study was shown by the name of the lead author and year of publication. The summary incidences were also shown in the figure. Plots are arranged as follows: (**A**) Sensitivity analysis of studies of incidence of all-grade cuSCC of BRAF inhibitor; (**B**) Sensitivity analysis of studies of incidence of high-grade cuSCC of BRAF inhibitor; (**C**) Sensitivity analysis of studies of incidence of all-grade cuSCC of dual BRAF/MEK inhibitors; (**D**) Sensitivity analysis of studies of incidence of high-grade cuSCC of dual BRAF/MEK inhibitors; (**E**) Sensitivity analysis of studies of RR of all-grade cuSCC; (**F**) Sensitivity analysis of studies of high-grade cuSCC.

Begg’s funnel plot and Egger’s test were performed to evaluate the publication bias of the eligible studies. Twenty-one studies investigating all-grade and high-grade cuSCC induced by single agent BRAF inhibitor yielded an Egger’s test score of *P* = 0.468 and *P* = 0.484, respectively, indicating the absence of publication bias in the studies (Figure 5). There were also no publication biases for incidence of dual BRAF/MEK inhibitors (*P* = 0.648 and *P* = 0.770, respectively). Results for publication bias from trials investigating RR were also shown in Figure 4 (*P* = 0.671 and 0.776 for RR of all-grade and high-grade cuSCC for single agent BRAF inhibitor versus dual BRAF/MEK inhibitors, respectively).

### Sensitivity analysis

**Figure 6:**
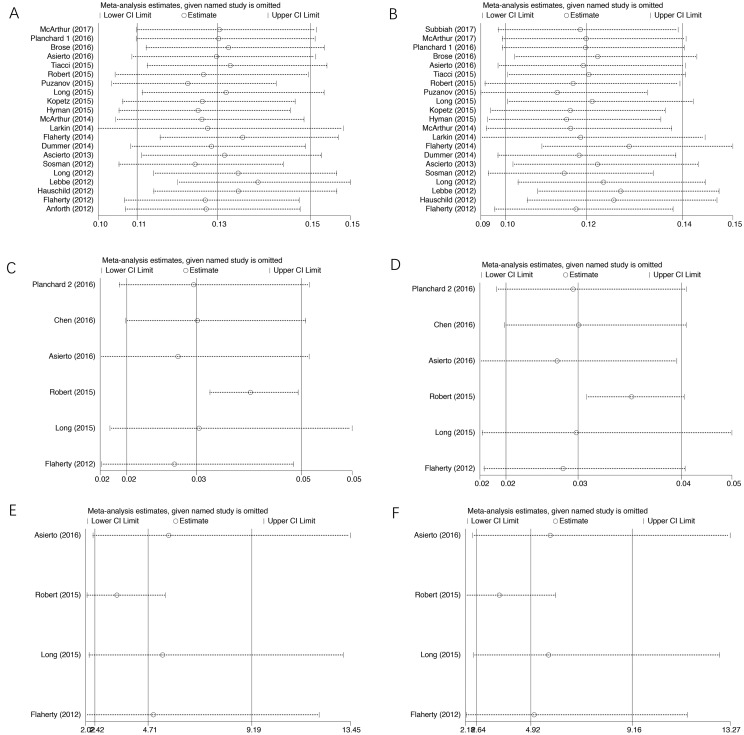
Sensitivity analysis for studies included in the meta-analysis Plots are arranged as follows: (**A**) Publication bias of studies of incidence of all-grade cuSCC of BRAF inhibitor; (**B**) Publication bias of studies of incidence of high-grade cuSCC of BRAF inhibitor; (**C**) Publication bias of studies of incidence of all-grade cuSCC of dual BRAF/MEK inhibitors; (**D**) Publication bias of studies of incidence of high-grade cuSCC of dual BRAF/MEK inhibitors; (**E**) Publication bias of studies of RR of all-grade cuSCC; (**F**) Publication bias of studies of high-grade cuSCC.

We did sensitivity analysis to examine the stability and reliability of pooled results by sequential omission of individual studies. The results indicated that the significance estimate of pooled incidences and RRs was not significantly influenced by omitting any single study (Figure 6).

## DISCUSSION

Activating *BRAF* mutations (most commonly *BRAF*^V600E^) are found in about 50% of melanomas, and lead to constitutive activation of BRAF and downstream MAPK signaling. The BRAF inhibitors vemurafenib [7] and dabrafenib [8] were first approved as single agents by the Food and Drug Administration (FDA) for the treatment of BRAF-mutated unresectable or metastatic melanoma.

In the RAS-RAF-MEK-ERK cell signaling pathway (MAPK pathway), MEK1 and MEK2 act downstream of BRAF. The most commonly described mechanism of resistance to BRAF inhibitors is reactivation of the MAPK pathway [9]. Thus, it has been suggested that combining BRAF inhibitor with inhibitors of other important molecules in the MAPK pathway (e.g. MEK) would overcome such resistance. Trametinib and cobimetinib are the two MEK inhibitors currently FDA-approved for melanoma treatment. The combination therapy increases apoptosis and delays the onset of resistance [10]. BRAF plus MEK-targeted drugs have outperformed BRAF inhibitor monotherapy in randomized clinical trials on BRAF-mutated melanoma [11–13]. The combination has become a new standard of treatment for BRAF-mutant advanced melanoma.

Cutaneous toxicity is observed in 92–99% of patients on BRAF inhibitor monotherapy [14], with the most common AE being the development of hyperproliferative epidermal neoplasms. Therapy with vemurafenib or dabrafenib has resulted in the frequent development of verrucal keratosis, plantar hyperkeratosis, Grover’s disease, actinic keratosis, and cutaneous squamous cell carcinoma [15]. As the use of novel agents has increased, so have the cutaneous toxicities associated with these medications. Development of cuSCC is due to RAF inhibition of wild-type BRAF cells, together with oncogenic RAS mutations present in photodamaged skin. Cutaneous SCC occur with BRAF inhibitor monotherapy because of paradoxical activation of the MAPK pathway in keratinocytes [16]. This can be blocked by the addition of a MEK inhibitor, thereby explaining the numerically lower incidence of these adverse events in patients receiving dual therapy.

We performed a meta-analysis to determine the incidence and relative risk of cuSCC among patients treated with BRAF inhibitor or dual BRAF/MEK inhibitors. To the best of our knowledge, this is the first meta-analysis evaluating the incidence and relative risk of cuSCC associated with BRAF inhibitor or dual BRAF/MEK inhibitors. In this comprehensive meta-analysis, prospective clinical trials and expanded access programs were included. The present meta-analysis has combined 24 publications including 5 phase 3 randomized controlled trials, 17 phase 2 trials, and 2 phase 4 trials.

Our meta-analysis results demonstrated that BRAF inhibitor is associated with an increased incidence and relative risk of developing cuSCC compared with dual BRAF/MEK inhibitors. The overall incidence of all-grade and high-grade cuSCC was 12.5% (95% CI: 10.8–14.6%) and 11.6% (95% CI: 9.8–13.8%) in single agent BRAF inhibitor, respectively. Subgroup analysis and meta-regression showed that no significant difference was detected between specific drug used (vemurafenib versus dabrafenib), melanoma versus non-melanoma, different study design in terms of all-grade and high-grade incidence of cuSCC. Dual BRAF/MEK inhibitors have an incidence of all-grade and high-grade cuSCC of 3.0% (95% CI: 2.0–4.5%) and 2.8% (95% CI: 1.9–4.0%), respectively. The relative risks of cuSCC of BRAF inhibitor compared to dual BRAF/MEK inhibitors were increased for all-grade and high-grade.

Combined BRAF and MEK inhibition reduces the incidence of cuSCC compared with BRAF monotherapy [17, 18], which was also confirmed by our meta-analysis. While some other adverse events (diarrhea, nausea, vomiting, photosensitivity reactions, increased creatine kinase levels, chorioretinopathy) occurred with a numerically higher incidence in patients receiving cobimetinib plus vemurafenib than in patients receiving vemurafenib alone, although these adverse events were mostly of grade 1 or 2 severity. The age of cuSCC is more prevalent in patients of 49 years or older, with a median age of 62 [14, 19, 20]. The median time to cuSCC/KA presentation is 8 weeks for vemurafenib, and 16 weeks for dabrafenib [21]. Malignant skin lesions can arise during therapy, requiring prompt dermatologic recognition and treatment in order to improve patient outcome. CuSCC are most often treated with a deep shave biopsy with electrodessication and curettage, or aggressive and frequent cryotherapy. NCCN and ESMO guidelines do not recommend single-agent BRAF therapy currently, while combination therapy is the optimal treatment option. However, in some countries such as China, considering the availability of drugs, high expenses and relatively low incidence of cuSCC in Asian population, domestic experts still consider single-agent BRAF inhibitor as standard first-line treatment. Furthermore, there are some studies investigating the suitable patient population for combination therapy.

Despite the size of this meta-analysis, our study has some limitations. First, confounding variables at the patient level, such as comorbidities, age and previous drug exposure could not be incorporated into the analysis. Secondly, a continuity correction of 0.5 subjects with an event was used, which may have slightly overestimated the actual event rate of individual trials. Thirdly, there was considerable heterogeneity among the primary studies. Finally, we did not have access to patient-level data.

In summary, our meta-analysis is the first study to systematically estimate the incidence of cuSCC associated with BRAF inhibitor. The relative risks of cuSCC of BRAF inhibitor compared with dual BRAF/MEK inhibitors were increased for nearly five-fold all-grade and high-grade. Close monitoring can help to avoid unnecessary dose reduction or treatment discontinuation and identify situations when treatment cessation is truly needed. These results would provide important information for clinicians who use BRAF inhibitor to treat patients.

## MATERIALS AND METHODS

### Search strategy and study selection

We systematically searched Pubmed, Embase and the Cochrane Database (up to June 2017) using various combinations of the terms: (“vemurafenib” or “PLX4032” or “Zelboraf”; or “dabrafenib” or “GSK2118436” or “Tafinlar”), and (“cancer” or “carcinoma” or “tumor” or malignancy” or “neoplasia”) and (“clinical trial” or “prospective trials” or “randomized controlled trial”). Furthermore, the American Society of Clinical Oncology (ASCO) abstracts database of the annual meetings was also searched. Additionally, we searched the clinical trial registration website (http://www.clinicaltrials.gov) to obtain information on the registered prospective trials.

Relevant clinical trials that met the following criteria were included: (1) prospective clinical trials in patients diagnosed with malignancy; (2) participants assigned to treatment with single agent BRAF inhibitor (vemurafenib or dabrafenib), or BRAF inhibitor in combination with MEK inhibitor (cobimetinib or trametinib); (3) the search was restricted to clinical trials and articles published in English; (4) events or event rate and sample size available for cuSCC, and (5) if multiple publications of the same trial were retrieved or if there was a case mix between publications, only the most informative one was included. Trials with relatively small number of patients (less than 20) were excluded. Phase I studies were excluded because of the different drug dosages and the relatively small number of patients on these trials. Review articles, irrelevant topics, case reports, and animal experimental studies were excluded. Abstracts of all candidate articles were read by two independent readers (LP and XY). Articles that could not be categorized based on title and abstract alone were retrieved for full-text review. Disagreements were resolved by consensus between the two readers.

### Study selection

Data abstraction was conducted independently by two investigators (LP and XY). Cutaneous SCC was extracted from the safety profile in each trial. Clinical endpoints were obtained from the safety profile of each clinical trial. In selected clinical trials, cuSCC was recorded according to the NCI-CTCAE versions 4.0. The CTCAE version 4.0 describes the grading as follows: grade I, asymptomatic or mild symptoms; clinical or diagnostic observations only; intervention not indicated; grade II, moderate; minimal, local or noninvasive intervention indicated; limiting age-appropriate instrumental ADL (activity of daily living); grade III, severe or medically significant but not immediately life-threatening; hospitalization or prolongation of existing hospitalization indicated; disabling; limiting self-care ADL; and grade IV, life-threatening consequences; urgent intervention indicated. We included all incidences of cuSCC of grade 1 or above in our analysis.

### Data analysis

The publications and data were reviewed and extracted by two independent investigators (LP and XY). The relevant information of each study including: (1) article or publication information, such as first author’s name, year of publication, etc.; (2) patient characteristics, such as diagnosis, age, gender, etc.; (3) study designation information, such as phase, total sample size, sample size per arm; (4) information about treatment, such as treatment approach, drug used; (5) events of cuSCC from the safety and toxicity profile and so on were carefully extracted, and they were recorded to a data collection form and then entered into an electronic database. If data from any of the above categories were not reported in the study, items were treated as “NR (not reported)”. Authors of the primary studies were not contacted for additional or unreported information.

### Statistical analysis

We derived the proportion and calculated the 95% confidence interval (CI) of patients with all-grade and high-grade events of cuSCC from each study. To calculate the pooled incidence, an inverse variance statistical method was used. For randomized controlled studies, we also calculated and compared the RRs of low grade and high-grade cuSCC. For one study that reported zero events in the control arm, we applied the classic half-integer correction to calculate the RR and variance [22]. RR > 1 indicated more cuSCC events in single agent BRAF inhibitor treatment arm; and vice versa.

The χ^2^-based Q statistic was applied to determine the heterogeneity between selected studies [23]. And the heterogeneity was considered to be statistically significant when heterogeneity < 0.10 or *I*^2^ > 50%. If heterogeneity existed, data was analyzed using a random-effects model, otherwise, a fixed-effects model was used.

To test for variation in incidence estimates by other factors, we conducted a meta-regression analysis. To explore the possible reasons for heterogeneity, we also conducted subgroup analyses by underlying malignancy, specific drug used, and different trial design. A two-sided *P* value of < 0.05 was considered statistically significant. The presence of publication bias was evaluated by using Begg’s and Egger’s tests [24, 25]. To assess the stability of results, sensitivity analysis was carried out by sequential omission of individual studies. All of the calculations were performed by STATA version 14.0 (Stata Corporation, College Station, TX).
